# The Interaction Between the Ventrolateral Preoptic Nucleus and the Tuberomammillary Nucleus in Regulating the Sleep-Wakefulness Cycle

**DOI:** 10.3389/fnins.2020.615854

**Published:** 2020-12-14

**Authors:** Juan Cheng, Fang Wu, Mingrui Zhang, Ding Ding, Sumei Fan, Guihai Chen, Jin Zhang, Liecheng Wang

**Affiliations:** ^1^Department of Physiology, School of Basic Medical Sciences, Anhui Medical University, Hefei, China; ^2^Teaching and Research Office of Physiology, School of Basic Medical Sciences, Anhui Medical College, Hefei, China; ^3^The Affiliated Chaohu Hospital, Anhui Medical University, Hefei, China; ^4^Department of Neurology, Second Affiliated Hospital of Nanjing Medical University, Nanjing, China

**Keywords:** VLPO, TMN, L-glutamate, bicuculline, GABA_*A*_-receptor, HRH_1_, sleep-wake circuitry

## Abstract

The ventrolateral preoptic nucleus (VLPO) in the anterior hypothalamus and the tuberomammillary nucleus (TMN) in the posterior hypothalamus are critical regions which involve the regulation of sleep-wakefulness flip-flop in the central nervous system. Most of the VLPO neurons are sleep-promoting neurons, which co-express γ-aminobutyric acid (GABA) and galanin, while TMN neurons express histamine (HA), a key wake-promoting neurotransmitter. Previous studies have shown that the two regions are innervated between each other, but how to regulate the sleep-wake cycle are not yet clear. Here, bicuculline (Bic), a GABA_*A*_-receptor antagonist, L-glutamate (L-Glu), an excitatory neurotransmitter, and triprolidine (Trip), a HA_1_ receptor (HRH_1_) inhibitor, were bilaterally microinjected into TMN or VLPO after surgically implanting the electroencephalogram (EEG) and electromyography (EMG) electrode recording system. Microinjecting L-Glu into VLPO during the night significantly increased the NREM sleep time, and this phenomenon was weakened after selectively blocking GABA_*A*_ receptors with Bic microinjected into TMN. Those results reveal that VLPO neurons activated, which may inhibit TMN neurons inducing sleep via GABA_*A*_ receptors. On the contrary, exciting TMN neurons by L-Glu during the day, the wakefulness time was significantly increased. These phenomena were reversed by blocking HRH_1_ with Trip microinjected into VLPO. Those results reveal that TMN neuron activating may manipulate VLPO neurons via HRH_1_, and induce wakefulness. In conclusion, VLPO GABAergic neurons and TMN histaminergic neurons may interact with each other in regulating the sleep-wake cycle.

## Introduction

The sleep-wake cycle is controlled by homeostasis and circadian rhythm, which regulates the amount, and the time of sleep, respectively (Borbely, 1982). The inhibitory relationship between sleep and wakefulness systems work as a trigger for the rapid conversion of sleep and wakefulness in the form of a positive feedback-loop (Wang et al., 2013). It is believed that GABAergic neurons in the ventral lateral hypothalamus (VLPO) and central preoptic region are the basis for the occurrence and maintenance of sleep (Sherin et al., 1996; Saper et al., 2010). Extracellularly electrophysiological recording results show that VLPO neurons have more activation during sleep, and the firing rate significantly increased during paradoxical sleep (Koyama and Hayaishi, 1994). Chemoactivating and photoactivating galanin-expressing neurons promoted total sleep time (TST), while photoinhibiting galanin-expressing neurons decreased NREM sleep (Kroeger et al., 2018). Lesions of the VLPO reduced sleep time and caused insomnia in cats and rats (Nauta, 1946; McGinty and Sterman, 1968; Lu et al., 2000). More than 85% of the neurons in the VLPO region are GABAergic neurons, which co-express the inhibitory neurotransmitters GABA and galanin (Sherin et al., 1996, 1998). VLPO neurons send axons to many regions that are implicated in the regulation of wakefulness, including the locus coeruleus (LC), median raphe nuclei, and the tuberomammillary nucleus (TMN) (Saper et al., 2010; Chung et al., 2017).

In the brain, the histaminergic (HAergic) neurons only gathered in the TMN. During the sleep-wake cycle, compared to non-rapid eye movement (NREM) sleep, the firing rate significantly increased during the awakening period, while they were almost silenced during rapid eye movement (REM) sleep (Sakai et al., 2010). TMN projects to almost the whole brain, and extraordinarily has highly dense innervation to the VLPO, the basal forebrain and the amygdala (Brown et al., 2001). Microinjection HA to VLPO can significant increase the locomotor activity of rats, also the electrophysiological experiments showed that HAs can inhibit the activity of VLPO neurons, the membrane potential hyperpolarized and firing rate were significantly decreased (Liu et al., 2010; Cheng et al., 2018).

Therefore, we predicted that the TMN and VLPO might inhibit each other, by which VLPO neurons release galanin and/or GABA at its terminal in TMN, and TMN neurons release HA and/or GABA in VLPO to maintain the balance of the sleep-wakefulness system. Here, we focus on the innervation of the two regions and the role of the neurotransmitters in the transition and maintenance of the sleep-wake cycle rhythm. In order to further explore and interpret the mechanisms in regulating sleep between VLPO and TMN, we inject cannula excitatory neurotransmitters L-glutamate (L-Glu), and GABA_*A*_ receptors antagonist bicuculline (Bic), and HRH_1_ antagonist triprolidine (Trip) in either VLPO or TMN, and *in vivo* recording by EEG and EMG observed the variations in rat sleep-wakefulness cycles. We found that the NREM sleep time was significantly increased after L-Glu was injected into VLPO, and this phenomenon was weakened after selectively blocking GABA_*A*_ receptors in TMN. Furthermore, both REM and NREM sleep time decreased after the excited TMN neurons, and these phenomena were reversed by blocked HRH_1_ in VLPO. Those results indicated that TMN histaminergic neurons and VLPO GABAergic neurons may interact with each other in regulating the sleep-wake cycle.

## Results

### Activating VLPO Neurons Decreased Wakefulness and Increased NREM in Rats

**FIGURE 1:**
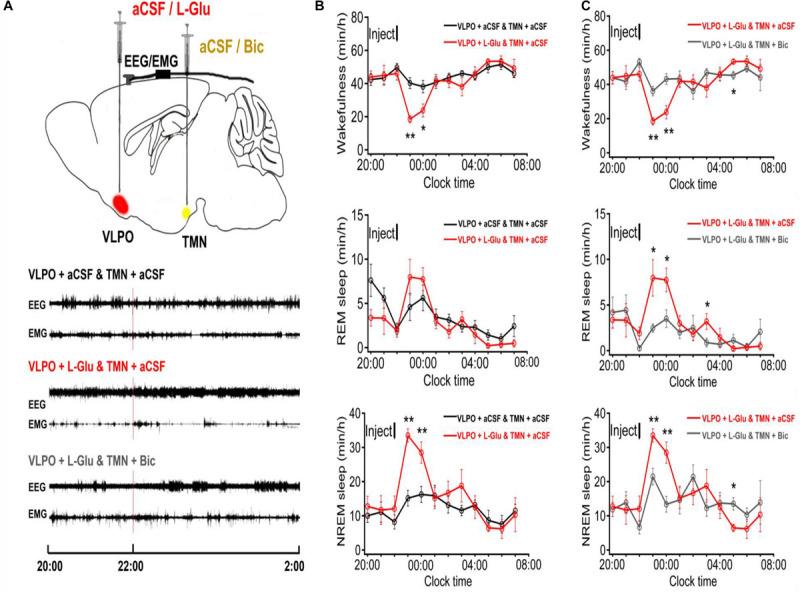
The sleep-wakefulness cycle was influenced by injecting L-Glu into VLPO which could be blocked by injecting Bic into TMN. **(A) Top:** Schematic drawing of the location of drug injection, or aCSF into the VLPO/TMN of a rat (Red: aCSF/L-Glu into VLPO, Yellow: aCSF/Bic into TMN). **Bottom:** Representative traces of EEG and EMG with drug injected. Drugs were applied at 22:00 (red line), and PSG recording started from 20:00 and lasted for 24 h. **(B)** Time-course changes of the sleep-wakefulness cycle in rat administration with L-Glu into VLPO. **(C)** Time-course changes of the sleep-wakefulness cycle in rat administration with L-Glu into VLPO and Bic into TMN. Data were presented as mean ± *SEM*. The paired *t*-test was used in the statistical comparisons of two groups. *p < 0.05, **p < 0.01 is compared between two groups at the same time. The arrow shows the time of microinjection.

A larger number of sleep-promoting neurons in VLPO were identified and innervated with the wake promoting system, and released inhibitory neurotransmitters (GABA) at its terminal, including TMN (Sherin et al., 1996, 1998). In order to investigate the effect of VLPO on the sleep-wake cycle, we microinjected aCSF (1 μl) into VLPO and TMN in a part of rats at 10:00–10:20 as the night control group, and microinjected L-Glu (L-Glu, 5 mmol/L with 1 μl) into VLPO and aCSF into TMN in another part of rats. Compared with the night control group (TMN + aCSF and VLPO + aCSF), the wakefulness time at the 2nd and 3rd h, after L-Glu was injected into VLPO, decreased about 54.0% (*p* < 0.01) and 37.6% (*p* < 0.05), respectively (Figure 1B top). The REM sleep time on the 2nd and the 3rd h after L-Glu was injected into VLPO increased about 73.1% and 37.6%, respectively (Figure 1B middle). However, the difference was not significant in REM sleep time. The NREM sleep time at the 2nd and 3rd h after L-Glu was injected into VLPO increased about 121.3% (*p* < 0.01) and 75.3% (*p* < 0.01), respectively (Figure 1B under).

**FIGURE 2:**
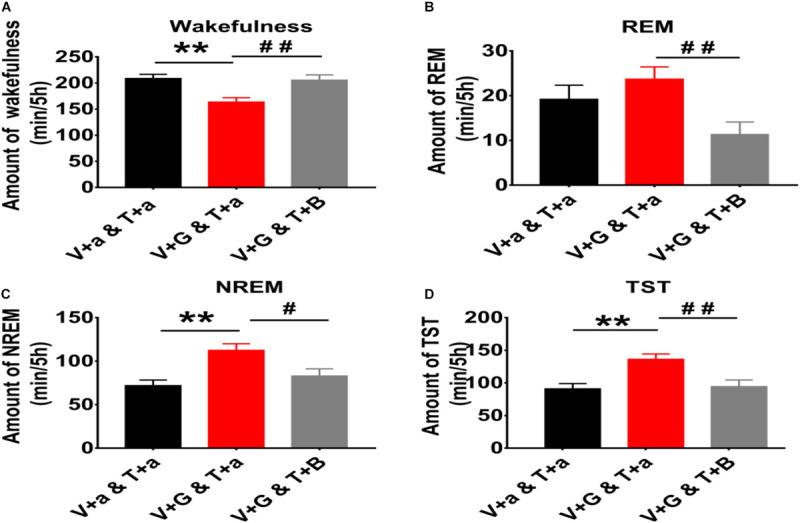
The 5 h cumulative amount of wakefulness, REM and NREM sleep, and TST after administration of aCSF or drugs into the TMN and VLPO of rats. **(A)** The 5 h cumulative amount of wakefulness. **(B)** The 5 h cumulative amount of REM sleep. **(C)** The 5 h cumulative amount of NREM sleep. **(D)** The 5 h cumulative amount of TST. Data were presented as mean ± *SEM*. The paired *t*-test was used in the statistical comparisons of two groups. Compared with the night group of VLPO + aCSF and TMN + aCSF group, **p < 0.01. Compared with the night group of VLPO + L-Glu and TMN + aCSF group, ^#^*p* < 0.05, ^##^*p* < 0.01. V, VLPO; T, TMN; G, L-Glu; B, Bic; a, aCSF.

The cumulative amount of wakefulness, REM sleep, NREM sleep, and TST in the next 5 h after injected L-Glu into VLPO were calculated. Compared with the night control group, the amount time of NREM sleep and TST after L-Glu was injected into VLPO increased about 56.4% (112.61 min ± 7.37 vs. 71.99 min ± 6.34, *p* < 0.01) and 49.5% (136.29 min ± 8.16 vs. 91.17 min ± 7.87, *p* < 0.01), meanwhile, the wakefulness time decreased by about 21.6% (163.71 min ± 8.17 vs. 208.83 min ± 7.89, *p* < 0.01), respectively (Figure 2). Those results indicate that excited VLPO neurons can decrease wakefulness and increase NREM at night in rats.

### Activating VLPO Neurons and Blocking GABA_*A*_ Receptors in TMN Significantly Affected the Sleep-Wakefulness Cycle in Rats

We designed to excite VLPO by L-Glu and inhibit TMN by Bic (0.1 mmol/L with 1 μl) in a rat, and analyzed the sleep-wakefulness cycle variation of rats. Compared with the night group of VLPO + L-Glu and TMN + aCSF, the wakefulness time at the 2nd and 3rd h after Bic injected into TMN was increased about 94.7% (*p* < 0.01) and 81.5% (*p* < 0.01), respectively (Figure 1C top). The REM sleep time at the 2nd and 3rd h after Bic was injected into TMN was reduced by 69.0% (*p* < 0.05) and 54.8% (*p* < 0.05), respectively (Figure 1C middle). The NREM sleep time at the 2nd and 3rd h after Bic was injected into TMN was decreased about 36.0% (*p* < 0.01) and 53.1% (*p* < 0.01), respectively (Figure 1C under).

The 5 h cumulative amounts of wakefulness, REM and NREM sleep times were calculated. Compared with the night group of VLPO + L-Glu and TMN + aCSF, the amount of wakefulness after Bic was injected into TMN increased about 25.6% (163.71 min ± 8.17 vs. 205.56 min ± 10.12, *p* < 0.01). Meanwhile the amount of REM sleep, NREM sleep and TST after Bic was injected into TMN decreased about 52.1% (23.67 min ± 2.78 vs. 11.33 min ± 2.78, *P* < 0.01), 26.2% (112.61 min ± 7.37 vs. 85.09 min ± 8.09, *p* < 0.05), and 30.7% (136.29 min ± 8.16 vs. 94.42 min ± 10.12, *p* < 0.01), respectively (Figure 2). Those results indicate that the effect of L-Glu exciting VLPO neurons on sleep-wakefulness at night can be inhibited by blocking GABA_*A*_ receptors in TMN in rats.

### Excited TMN Neurons Increased Wakefulness Time and Decreased REM and NREM Sleep Time in Rats

**FIGURE 3:**
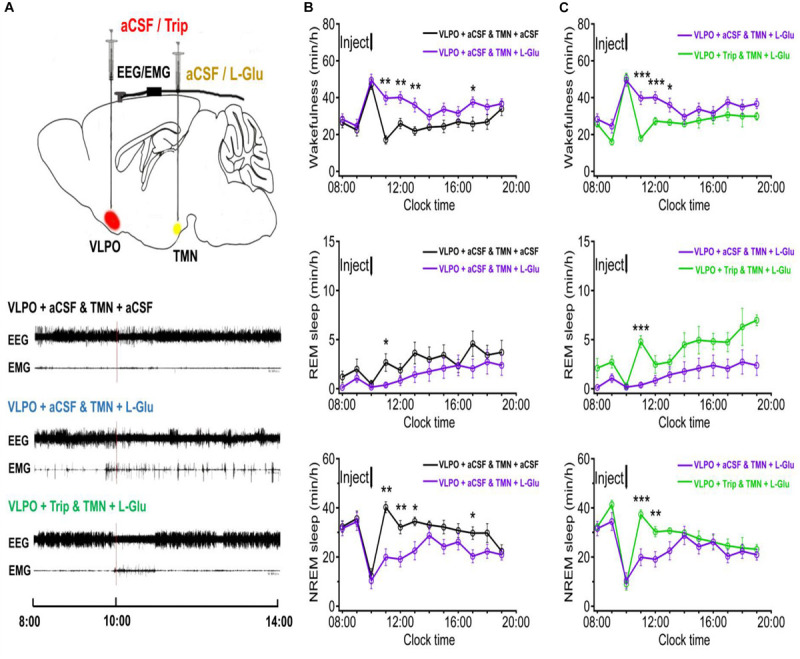
The 5 h cumulative amounts of wakefulness, REM and NREM sleep, and TST after administration of aCSF or drugs into the TMN and VPO of rats. **(A)** The 5 h cumulative amount of wakefulness. **(B)** The 5 h cumulative amount of REM sleep. **(C)** The 5 h cumulative amount of NREM sleep. **(D)** The 5 h cumulative amount of TST. Data were presented as mean ± *SEM*. The paired *t*-test was used in the statistical comparisons of two groups. Compared with the day group of VLPO + aCSF and TMN + aCSF, **p < 0.01; Compared with the day group of VLPO + aCSF and TMN + L-Glu, ^#^*p* < 0.05, ^##^*p* < 0.01. V, VLPO; T, TMN; G, L-Glu; B, Bic; a, aCSF.

**FIGURE 4:**
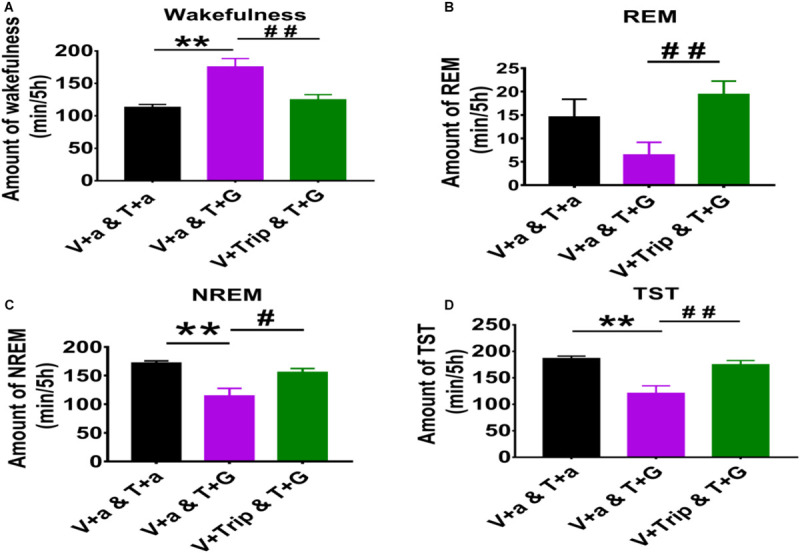
The sleep-wakefulness cycle was influenced by injecting L-Glu into TMN which could be blocked by injecting Trip into VLPO. **(A)** Top: Schematic drawing of the location of drug injection, or aCSF into the VLPO/TMN of a rat (Red: aCSF/Trip into VLPO, Yellow: aCSF/L-Glu into TMN). Bottom: Representative traces of EEG and EMG with drug injected. Drugs were applied at 10:00 (red line), and polysomnography recording started from 08:00 and lasted for 24 h. **(B)** Time-course changes of the sleep-wakefulness cycle in rat administration with L-Glu into TMN. **(C)** Time-course changes of the sleep-wakefulness cycle in rat administration with L-Glu into TMN and Trip into VLPO. Data were presented as mean ± *SEM*. The paired *t*-test was used in the statistical comparisons of two groups. *p < 0.05, **p < 0.01, ***p < 0.001 is compared between two groups at the same time. The arrow shows the time point of microinjection.

In order to figure out whether the excited TMN neurons would reduce the sleep time and increase the wakefulness time, we stereotaxically implanted cannulas and microinjected excitatory neurotransmitters L-Glu to TMN at 10:00–10:20. The representative traces of EEG and EMG of the 4 day groups after either injection of aCSF or drugs are shown in Figure 3B. Microinjecting L-Glu (5 mmol/L with 1 μl) into TMN increased the wakefulness time of rats at the 2nd h and lasted 3 h (Figure 4B). Compared with the day group of VLPO + aCSF and TMN + aCSF group, the wakefulness time after L-Glu was injected into TMN at the 2nd, 3rd, and 4th h was increased about 132.2% (*p* < 0.01), 54.3% (*p* < 0.01), and 64.6% (*p* < 0.01), respectively (Figure 4B upper). The REM sleep time after L-Glu was injected into TMN at the 2nd, 3rd, and 4th h decreased about 85.9% (*p* < 0.05), 55.9% and 60.6%, respectively. However, there were no significant differences after L-Glu was injected into TMN at the 3rd and 4th h (Figure 4B middle). The NREM sleep time after L-Glu was injected into TMN at the 2nd, 3rd, and 4th h was reduced to about 50.5% (*p* < 0.01), 40.6% (*p* < 0.01), and 34.5% (*p* < 0.05), respectively (Figure 4B under).

Compared with the day group of VLPO + aCSF and TMN + aCSF group, the 5 h cumulative amount of wakefulness time after L-Glu was injected into TMN was increased about 35.0% (113.35 min ± 4.32 vs. 179.00 min ± 13.91, *p* < 0.01). Meanwhile, the 5 h cumulative amount of REM sleep time, NREM sleep time and TST decreased about 55.5% (14.57 min ± 3.79 vs. 6.48 min ± 2.68, *p* > 0.05), 33.4% (172.05 min ± 3.57 vs. 114.51 min ± 13.07, *p* < 0.01) and 35.2% (186.63 min ± 4.32 vs. 120.99 min ± 13.91), respectively (Figure 3). Those results indicate that L-Glu excited TMN neurons which increased wakefulness and decreased NREM during the day.

### Activating TMN Neurons and Blocking HRH_1_ Receptors in VLPO Significantly Affected the Sleep-Wakefulness Cycle in Rats

It has been proven that the histaminergic neurons of TMN can project to the VLPO (Sakai et al., 2010; Chung et al., 2017). Here, we microinjected triprolidine (Trip, 0.5 μmol/L with 1 μl), a HRH_1_ blocker, into VLPO to block the histaminergic afference from TMN. Compared with the day group of VLPO + aCSF and TMN + L-Glu, the wakefulness time at the 2nd, 3rd, and 4th h after Trip was injected into VLPO was reduced by about 54.9% (*p* < 0.01), 32.1% (*p* < 0.01), and 26.1% (*p* < 0.05), respectively (Figure 4C upper). The REM sleep time at the 2nd, 3rd, and 4th h after Trip was injected into VLPO increased about 1175.4% (*p* < 0.01), 196.5% (*p* > 0.05), and 89.7% (*p* > 0.05), respectively (Figure 4C middle). The NREM sleep time at the 2nd, 3rd, and 4th h after Trip was injected into VLPO increased by about 87.5% (*p* < 0.01), 58.8% (*p* < 0.05), and 36.0%(*p* > 0.05), respectively (Figure 4C under).

Compared with the day group of VLPO + aCSF and TMN + L-Glu group, the 5 h cumulative amount of wakefulness time after Trip was injected into VLPO was decreased by 30.2% (179.00 min ± 13.91 vs. 125.03 min ± 7.68, *p* < 0.01), meanwhile, the 5 h cumulative amount of REM sleep, NREM sleep, and TST were increased about 199.3% (6.48 min ± 2.68 vs. 19.40 min ± 2.85, *p* < 0.01), 35.9% (114.51 min ± 13.07 vs. 155.57 min ± 6.80, *p* < 0.05), and 44.6% (120.99 min ± 13.91 vs. 174.97 min ± 7.66, *p* < 0.01), respectively (Figure 3). Those results indicate that the effect of exciting TMN neurons on sleep-wakefulness at day can be inhibited by blocking HRH_1_ receptors in VLPO in rats.

## Discussion

The sleep-promoting system is mainly composed of the preoptic area and adjacent basal forebrain. Sleep deprivation might induce the brain and cognitive function disorder, such as learning and memory. Recently, studies have shown that light-induced SWS (slow wave sleep) might have strongly enhanced the process in memory consolidation (Lu et al., 2018). Sleep inducing neurons in the preoptic area are mainly located in the VLPO, as many *c-fos*-positive neurons found after sleep to have recovered following sleep deprivation (Zhang et al., 2015). Neurons in the VLPO synthesized and released the inhibitory neurotransmitter GABA, and their fibers terminal can innervation to 5-HT neurons in dorsal raphe nucleus, blue-spot norepinephrine neurons, cholinergic neurons in lateral tegmental nucleus, and histaminergic neurons in TMN. The inhibitory effect of the sleep-promoting neurons innervated from VLPO can reduce the sleep-promoting effects of HA neurons and other key arousal regions, inducing and maintaining sleep (Sherin et al., 1996; Saper et al., 2010; Chung et al., 2017; Scammell et al., 2017).

For the arousal system, polysomnography, pharmacological, and tracing studies showed that different functional cell groups with different terminal projections might modulate the sleep-wake switch though various signaling pathways, such as histaminergic, glutamatergic, orexinergic, GABAergic pathways, and so on (Liu et al., 2010; Eban-Rothschild and de Lecea, 2017; Scammell et al., 2017; Schöne and Burdakov, 2017). Among them, TMN is an area where histaminergic neurons are concentrated in the cell body of the central nervous system, and can project the orexinergic neurons on the lateral hypothalamus and the basal forebrain (Eriksson et al., 2001; Schöne and Burdakov, 2017).

As an excitatory neurotransmitter, L-Glu can selectively excite neurons via N-methyl -D-aspartate (NMDA) receptors, which play important roles in synaptic plasticity, synaptic transmission and neuron degeneration (Swaminathan et al., 2019). The administration of kainite, a type of L-Glu receptor antagonist, into the nucleus ceruleus in the rat can induce a significant increase in REM sleep, however, damage to the preoptic area with kainite in the rat can decrease sleep and increase wakefulness (John et al., 1994; Onoe and Sakai, 1995; Vataev et al., 2013). In our study, we found that microinjecting L-Glu into VLPO can inhibit wakefulness and increase NREM sleep, but has no significant effect on REM sleep. Therefore, this result infers that the injection L-Glu in VLPO might excite sleep-promoting neurons and maintain NREM sleep.

We found the microinjection of Bic (Ramshini et al., 2019), a GABA_*A*_ receptor specific antagonist, into TMN has no significant effect on the sleep-wakefulness cycle. Bic can directly work on histaminergic neurons to increase its firing rate (Haas et al., 2008), and the perfusion of Bic into the hypothalamus can increase the expression of HAs in the nucleus accumbens and the prefrontal cortex (Cenni et al., 2006; Giannoni et al., 2009). Thus, we microinjected Bic to TMN in the early night. During the wakefulness state, the release of inhibitory neurotransmitters at the terminal projection of VLPO were decreased, while histaminergic neurons in TMN are particularly activating, and the firing of histaminergic neurons is significantly higher than that during the sleep phase (Zeitzer et al., 2012; Valko et al., 2013), with up-regulation of c-*fos*-protein expression (Ko et al., 2003).

In this study, L-Glu microinjected into VLPO during the night significantly increased the NREM sleep time and decreased the wakefulness time. Those phenomena were weakened after selectively blocking GABA_*A*_ receptors by Bic microinjected into TMN. The phenomena were consistent with the previous results of injecting GABA_*A*_ receptor blockers into TMN and adjacent areas, which can block the sleep-inducing effects caused by central sedatives and anesthetics, such as pentobarbital, muscarine, and propofol (Nelson et al., 2002). Thus, it may be deduced that activated VLPO neurons can induce sleep, which may mainly inhibit TMN neurons via GABA_*A*_ receptors.

Bilateral microinjection HA into the basal forebrain of rats showed that dose-dependent wakefulness was increased and accompanied by the decreasing of NREM sleep, suggesting that HAs might induce wakefulness by relayed cholinergic neurons in the basal forebrain (Ramesh et al., 2004). Microinjection HAs into the VLPO can increase the activity rate of rats, and the electrophysiological experiment of isolated brain slices showed that HAs can inhibit the activity of VLPO neurons by over-polarizing the membrane potential (Liu et al., 2010). In this study, microinjection of L-Glu into TMN at day results in increasing wakefulness time and decreasing sleep time in rats, which was weakened when Trip, a HRH_1_ blocker, was injected into VLPO at the same time. These results reveal that activated TMN neurons might manipulate VLPO neurons via HRH_1_, and induce wakefulness.

In conclusion, our results indicate that activation of VLPO by L-Glu can promote sleep and weaken the transition to wakefulness. Moreover, inactivation of TMN by GABA_*A*_ antagonist can turnover those phenomena. On the contrary, exciting TMN neurons by glutamate receptor antagonist can promote wakefulness and weaken the transition to sleep, and, these phenomena can be reversed by blocking HRH_1_ with Trip microinjected into VLPO. This relationship between TMN arousal and VLPO sleep-promoting pathways may produce the conditions for a flip-flop switch, which can generate rapid and complete transitions between waking and sleeping states, but certain types of neurons in VLPO and TMN participate and modulate the transition between REM, NREM, and wakefulness need to be further studied.

## Materials and Methods

### Animal Model

Adult male Sprague Dawley rats (SPF grade) weighing 270–290 g were used. All rats were housed in a free moving environment kept at room temperature (22–24°C), with the humidity maintained at 55% and 12 h of light/dark (light on 8:00–20:00 h, illumination intensity ≈ 100 lx). The sound insulation shielding and ventilated environment was kept separately, free to water and feeding. The animals in the experiment were kept strictly in accordance with the regulations of the People’s Republic of China on the management of experimental animals and the methods for quality management of experimental animals.

### Surgery and Implantations for *in vivo* Polysomnographic Recording

After anesthetized by pentobarbital (50 mg/kg, i.p.), EEG and EMG electrodes were implanted for polysomnographic recording (MP150, Data acquisition and analysis system, Biopac Ltd., United States), and two guide cannulas were bilaterally inserted into VLPO (AP: −0.36 mm; R: 1.00 mm; H: −7.50 mm) and TMN (AP: −4.20 mm; R: 1.10 mm; H: −7.70 mm) for drug application in rats. The microinjection cannulas for drugs were embedded at 2 mm above the VLPO or TMN regions in the brain. The recording electrodes for EEG recording were embedded at 1 mm in front of the coronal suture and herringbone stitch before 1 mm and side open 1 mm node installed on both sides of the midline skull, and recording electrodes for EMG were inserted into the bilateral neck muscle. Guide cannulas and recording electrodes were fixed to the skull surface with dental cement. Each animal needed 7 days for recovery in a sound proof recording room after surgery, then they were connected to an EEG/EMG recording cable and habituated for 3 days before polysomnographic recording.

### Grouping

Rats were randomly divided into day and night groups: the night group was composed of (1) VLPO + aCSF (artificial cerebrospinal fluid) and TMN + aCSF group (both TMN and VLPO are microinjected with aCSF (Cheng et al., 2018) containing (mM): 125 NaCl, 1.25 KCl, 25 NaHCO_3_, 1.25 KH_2_PO_4_, 25 D-Glucose, 2 CaCl_2_, 1 MgCl_2_, supplemented with 400 Na-pyruvate and 80 L-ascorbic acid, *n* = 8), The pH was adjusted to 7.25 with D-gluconic acid and osmolarity was adjusted to 290–300 mOsm with D-Glucose as necessary; (2) VLPO + L-Glu and TMN + aCSF group (Microinjection of aCSF and L-Glu into TMN and VLPO, respectively, *n* = 7); (3) VLPO + L-Glu and TMN + Bic group [microinjection of Bic (Sigma, St. Louis, MO, United States) and L-Glu (Sigma, St. Louis, MO, United States) into TMN and VLPO, respectively, *n* = 8]. The day group was composed of (1) VLPO + aCSF and TMN + aCSF group (both VLPO and TMN are microinjected with aCSF, *n* = 7); (2) VLPO + aCSF and TMN + L-Glu group (microinjection of aCSF and L-Glu into VLPO and TMN, respectively, *n* = 8); (3) VLPO + Trip and TMN + L-Glu group [microinjection of Trip (Sigma, St. Louis, MO, United States) and L-Glu into the VLPO and TMN, respectively, *n* = 7].

### Microinjection

The drugs (L-Glu, Bic, and Trip) of 1 μl were dissolved in aCSF. Each administration was 5 mmol/L L-Glu, 0.1 mmol/L Bic or 0.5 μmol/L Trip. Control groups were microinjected in aCSF. The microinjection was through a stainless steel guide cannula at an injection rate of 1 μl per min, and the needle was kept in the cannula for 1 min to prevent the physic liquor overflow. For the night group, the administration was performed at 22:00–22:20 for polysomnography (PSG) recording. For the day group, the administration was performed at 10:00–10:20 for PSG recording. Figures 1A, 4A present the schematic drawing of the location of drug or aCSF injection into the VLPO/TMN of the rat.

### Polysomnography Recording

Polysomnography recording (including EEG and EMG) was started 2 h before drug application at 20:00 or 08:00, and was sustained for 24 h. According to the PSG results, every 10 s were regarded as one epoch. The sleep-wake cycle is divided into wakefulness (W), non-rapid eye movement sleep (NREM), and rapid eye movement sleep (REM), each of which have distinct characteristics. W was characterized by high frequency and low amplitude waves of EEG and relatively high tone EMG; NREM sleep was characterized by low frequency, spindles, high amplitude and slow waves of EEG with significantly decreased EMG tone, and REM sleep was characterized by high frequency and low amplitude waves of EEG with a lack of EMG tone, except for occasional muscle twitches. The representative traces of EEG and EMG of the four night groups after either injection of aCSF or drugs were shown in Figure 1A. In our study, we counted the total sleep time (TST),which was composed of NREM sleep and REM sleep time.

### Statistical Analyses

GraphPad Prism 7 was used for statistical analyses. The experimental data was presented as mean ± *SEM*. The paired *t*-test was used in the statistical comparisons of the experimental data between the two groups, the line charts were performed in Igor pro (WaveMetrics, Portland, OR, United States), and those at *P* < 0.05 were considered as the level of significance.

## Data Availability Statement

The raw data supporting the conclusions of this article will be made available by the authors, without undue reservation.

## Ethics Statement

The animal study was reviewed and approved by the Institutional Animal Care Unit Committee of Anhui Medical University. Written informed consent was obtained from the owners for the participation of their animals in this study.

## Author Contributions

LW and JZ designed and supervised the research work. JC wrote the manuscript and revised the manuscript. JC, FW, and GC performed and analyzed the polysomnographic recording experiments. MZ, DD, and SF set up the recording system and breed rats. All authors contributed to the article and approved the submitted version.

## Conflict of Interest

The authors declare that the research was conducted in the absence of any commercial or financial relationships that could be construed as a potential conflict of interest.

## Supplementary Material

Supplementary Figure 1 VLPO and TMN injection sites in all the animals used in the experiments. **(A)** An example brain slice was obtained by frozen section which showing cannulae locations in the VLPO. **(B)** An example brain slice was obtained by frozen section which showing cannulae locations in the TMN. Scale bar = 100 μm.
