# Supplementary figures and images for: The Interaction Between the Ventrolateral Preoptic Nucleus and the Tuberomammillary Nucleus in Regulating the Sleep-Wakefulness Cycle
Source: Front Neurosci. 2020 Dec 14;14:615854. doi: 10.3389/fnins.2020.615854 (PMC7767984; doi:10.3389/fnins.2020.615854)

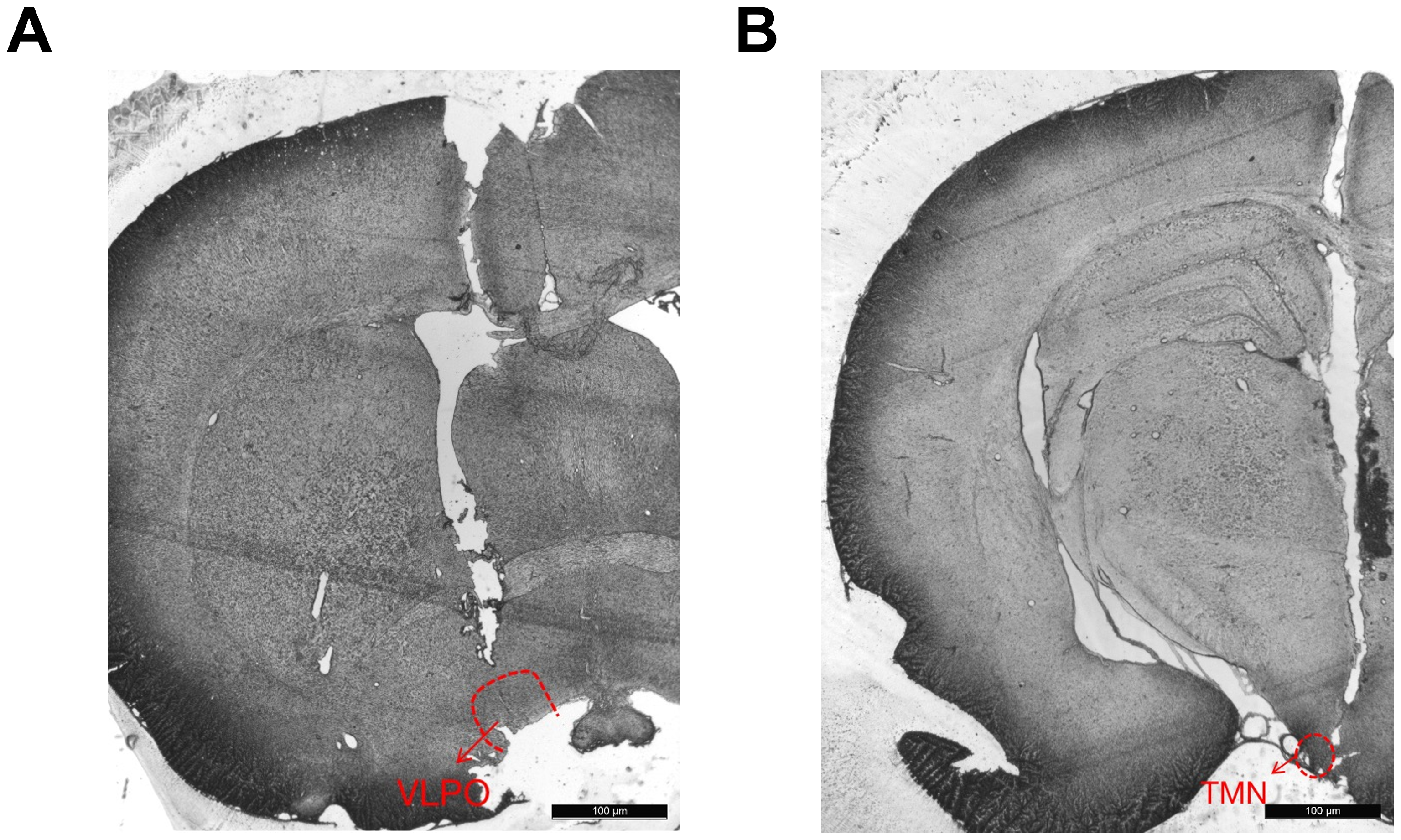

Supplement: Supplementary Figure 1 — VLPO and TMN injection sites in all the animals used in the experiments. (A) An example brain slice was obtained by frozen section which showing cannulae locations in the VLPO. (B) An example brain slice was obtained by frozen section which showing cannulae locations in the TMN. Scale bar = 100 μm. [file Image_1.TIF]
